# Medical students’ perspective: analysis of risk communication teaching in psychosocial and other medical departments

**DOI:** 10.1080/10872981.2020.1784375

**Published:** 2020-06-19

**Authors:** Mohamed Rifdhy Mohamed Riza, Jawwad Mihran Haider, Isaamuddin Alvi

**Affiliations:** Medical Student at St George’s, University of London, London, UK

**Keywords:** Medical education, medical students, risk communication, medical school, teaching, medical statistics

We read with great interest the study by Baessler et al. [1], which considers risk communication teaching in medical schools. We were impressed by the data processing and presentation by the authors, which essentially highlights the lack of both quantity and quality of risk communication teaching. Although this study was conducted at Heidelberg University Medical School, we believe the issue of lack of risk communication teaching applies worldwide. A similar study in the UK found that most clinicians ‘had not recognised the value of their undergraduate teaching in statistics and probability at the time’ [2]. We thank the authors for raising this paramount issue; as senior medical students having experienced risk communication teaching, we would like to explore and discuss certain aspects of the article in further detail.

The study showed that risk communication topics were only mentioned in 6.1% of PBL sessions compared to 34.8% of practical training sessions. Likewise, demonstration material used for risk communication was only used once in PBL sessions. Having been involved in such sessions, we have also noted the lack of teaching in risk communication. The use of PBL allows understanding and application of important medical concepts and, as medical students at a university adopting a PBL curriculum, we believe that PBL becomes particularly effective in appreciating more demanding and niche topics such as risk communication. Therefore, we wonder why more medical schools do not incorporate learning opportunities for risk communication. Whilst integrating risk communication into PBL does pose challenges, such as recruiting tutors with both clinical and statistical expertise and developing specific patient cases, we can learn from the various models employed by medical schools in Australia, which integrates statistical material into PBL sessions [3].

The authors make note of the fact that few questions were asked in sessions where risk communication was taught, indicating low interest in the subject amongst students and a ‘failure to induce discussion. A study conducted in the University of Makassar, Indonesia, delved into factors influencing questioning powers of students in class and found that, although interest in materials did affect students’ likelihood to ask questions, other factors such as personality characteristics of teachers, emotional state of the student and, particularly, being afraid of asking questions also contributed. Indeed, 72% of students felt afraid to ask questions in class, with many of them preferring to ask their friends instead or teachers face-to-face after class [4]. As medical students, we can attest to the feelings of intimidation which arise when asking questions during lectures. We propose that small designated question-and-answer sessions may enable students to feel more confident to ask questions and improve interactivity in sessions involving risk communication teaching.

Despite the contributions of the paper, we do question the need for statistical values to be taught in-depth repetitively. The research found ‘in 90% of these teaching sessions 16 out of 19 common statistical values … were only mentioned without further elaboration’. Whilst we appreciate that statistical values are important, the authors have noted correctly student engagement is low in these sessions. Interestingly, the authors have found ‘that traditional literature handouts were the most preferred choice of supplementary material’. We, therefore, suggest that a literature handout with explanations of the 19 common statistical values could be produced and given to aid students in these sessions, not only increasing engagement but also providing standardised teaching to reduce the levels of ‘statistical illiteracy’.

Academic staff could also observe lectures adding a different perspective to the delivery of lecture contents. This comparison could explain potential omissions, as seen with the 112 sessions where students ‘could not observe any risk communication teaching’, shedding light on positive avenues for reform in medical universities. Furthermore, we suggest that future studies should allow students to add free-text comments to evaluate risk communication teaching, as openly discussing the syllabus would be hugely beneficial in student understanding. A study carried out in South Africa found that qualitative course evaluations were a suitable alternative to quantitative surveys [5]. A more in-depth qualitative analysis would highlight which statistical values are not taught as well, as observers’ answers are limited to ‘mentioned’, ‘reflected upon’, ‘transferred to a clinical context’ and ‘set in practical context’.
